# Potential impact of new oral anticoagulants on the management of atrial fibrillation-related stroke in primary care

**DOI:** 10.1111/ijcp.12177

**Published:** 2013-04-28

**Authors:** K Harris, J Mant

**Affiliations:** Primary Care Unit, Department of Public Health & Primary Care, Strangeways Research Laboratory, University of CambridgeCambridge, UK

## Abstract

**Aim:**

Anticoagulant prophylaxis with vitamin K antagonists (such as warfarin) is effective in reducing the risk of stroke in patients with atrial fibrillation (AF). New oral anticoagulants have emerged as potential alternatives to traditional oral agents. The purpose of this review was to summarise the effectiveness and safety of rivaroxaban, dabigatran and apixaban in stroke prevention in patients with AF in phase III trials, evaluate their cost-effectiveness and consider the implications for primary care.

**Methodology:**

A literature search was performed between 2007 and 2012, selecting all phase III trials (ROCKET AF, RE-LY and ARISTOTLE) of new oral anticoagulants and relevant cost–benefit studies.

**Results:**

Evidence shows that all three agents are at least as effective as warfarin in the prevention of stroke and systemic emboli, with similar safety profiles. Cost–benefit studies of rivaroxaban and dabigatran further confirm their potential use as alternatives to warfarin in clinical practice. These observations may allow stratification of the general practice AF population, to help prioritise which patients may benefit from receiving a new oral anticoagulant.

**Conclusion:**

The clinical and economic benefits of the new oral anticoagulants, along with appropriate risk stratification, may enable a higher number of patients with AF to receive effective and convenient prophylaxis for stroke prevention.

**Review criteria**

MEDLINE searches were performed to include publications from 2007 to May 2012. One search used the MeSH terms ‘anticoagulants’, ‘atrial fibrillation’ and ‘clinical trial’, and a second search used the MeSH terms ‘anticoagulants’, ‘atrial fibrillation’ and ‘cost–benefit analysis’. All phase III trials of new oral anticoagulants and relevant cost-effectiveness publications were selected following review of titles and abstracts.

**Message for the clinic**

The new oral anticoagulants offer effective and convenient alternatives to warfarin for the prevention of stroke in patients with atrial fibrillation. These drugs are cost-effective, but their introduction into clinical practice may be a challenge because of higher total drug costs. Stratification of the general practice atrial fibrillation population by stroke risk and warfarin experience may aid physicians in prioritising who should receive treatment with these new agents.

## Introduction

Each year, approximately 110,000 people in England have a stroke, at a cost to the economy of around £7 billion (1). Atrial fibrillation (AF) is a known risk factor for stroke, imparting a fivefold increase in risk (2), and AF has a prevalence of around 1–2.5% (3,4). The risk of AF is strongly associated with old age, and prevalence rises to over 9% in people in their 80s (5).

Anticoagulation with warfarin substantially reduces the risk of stroke in patients with AF (6,7), but is associated with an increased risk of intracranial haemorrhage and other bleeding (8). Warfarin continues to be ‘underused’ compared with guideline-recommended care. In a study of health maintenance organisation patients in North Carolina, USA, less than 60% of patients with one or more risk factors for stroke and no contraindications were receiving warfarin (9). Another large study of patients with AF discharged from hospital found that only 64.6% of ideal candidates were prescribed warfarin (10). A review of 310 practice populations in the UK showed that only 27% of high-risk (CHADS_2_ score > 1) patients with AF not taking warfarin had a contraindication for its use (11). Low usage of warfarin is in part caused by high discontinuation rates. For example, in the Birmingham Atrial Fibrillation Treatment of the Aged (BAFTA) trial, a third of patients randomised to warfarin had stopped taking it after an average follow up of 2.7 years (12). In addition, physicians may not initiate warfarin therapy because of a fear of intracranial haemorrhage, especially in elderly patients (11,13). Warfarin may be associated with further complications in patients with comorbidities associated with haemorrhagic risk (13), and some patients are deterred by the need for regular coagulation monitoring and the risk of interactions with food and other drugs (14).

Until recently, alternatives to warfarin have been unsatisfactory. Antiplatelet agents, although easier to use, are significantly less effective (15), even when used in combination (16). However, three promising new oral anticoagulants (OACs) (17–19) have emerged: dabigatran etexilate, a direct thrombin inhibitor, and rivaroxaban and apixaban, which are both direct Factor Xa inhibitors. Both rivaroxaban and dabigatran are approved in the EU and USA, as well as in other countries, for stroke prevention in patients with AF. A decision by the US Food and Drug Administration on the use of apixaban in patients with AF has been delayed until 2013. There is also a potential fourth agent (edoxaban), for which a phase II dose-finding trial has been conducted (20). In this article, we review the evidence of the effectiveness and safety of the three agents for which phase III trials have been published, and consider their cost-effectiveness and the potential implications for practice in primary care.

## Methods

We searched MEDLINE using the MeSH terms ‘anticoagulants’ and ‘atrial fibrillation’ and ‘clinical trial’, limiting our search to publications from 2007 to May 2012. We then reviewed titles and abstracts to find all phase III trials of new OACs. We then ran a separate MEDLINE search using the MeSH terms ‘anticoagulants’ and ‘atrial fibrillation’ and ‘cost–benefit analysis’, again limiting our search to publications from 2007 to May 2012 and reviewing titles and abstracts to find relevant papers.

## Safety, effectiveness and side effect profile

The key findings from the trials that tested these drugs against warfarin are summarised in Tables 1 and 2. Direct cross-trial comparisons are problematic owing to differences in trial designs (i.e. open-label vs. double-blind), statistical analyses and baseline stroke risk in the study populations. Nonetheless, all three new OACs were at least as effective as warfarin in preventing strokes and systemic emboli, and within the context of the individual trials, apixaban and higher dose dabigatran were superior to warfarin (17–19). Furthermore, the risk of death was reduced by approximately 10% compared with warfarin (21). The main safety concern for anticoagulation therapy is an increased risk of bleeding. Rivaroxaban and higher dose dabigatran were associated with similar risks of major bleeding compared with warfarin, whereas apixaban and lower dose dabigatran were associated with lower risks (17–19). All three new OACs were associated with a lower risk of intracranial haemorrhage compared with warfarin (17–19).

**Table 1:** Overview of efficacy data from phase III trials investigating rivaroxaban, dabigatran or apixaban compared with warfarin for the prevention of stroke and systemic embolism in patients with AF

					Hazard ratio (intention to treat; 95% CI)		
					
Study	Method	Population	Mean CHADS_2_ score	Drug	Stroke (all types) or systemic embolism	Myocardial infarction	Deaths
ROCKET AF (18)	Double-blind, double-dummy, randomised, non-inferiority trial	14,264 patients with non-valvular AF with either a history of stroke, TIA or systemic embolus, or a CHADS_2_ score of ≥ 2 from 1178 participating sites in 45 countries. Mean age 73 years	3.5	Rivaroxaban 20 mg od*	0.88 (0.75–1.03) p < 0.001 for non-inferiority, p = 0.12 for superiority	0.81 (0.63–1.06) p = 0.12†	0.92 (0.82–1.03) p = 0.15
RE-LY (17)	Open-label, randomised, non-inferiority trial	18,113 patients with AF and risk factors for a stroke, from 951 centres in 44 countries. Mean age 71 years	2.1	Dabigatran 150 mg bid	0.66 (0.53–0.82) p < 0.001 for non-inferiority, p < 0.001 for superiority	1.38 (1.00–1.91) p = 0.048	0.88 (0.77–1.00) p = 0.051
				Dabigatran 110 mg bid	0.91 (0.74–1.11) p < 0.001 for non-inferiority, p = 0.34 for superiority	1.35 (0.98–1.87) p = 0.07	0.91 (0.80–1.03) p = 0.13
ARISTOTLE (19)	Double-blind, double-dummy, randomised, non-inferiority trial	18,201 patients with AF and ≥ 1 additional risk factor for stroke from over 1000 centres in 39 countries. Median age 70 years	2.1	Apixaban 5 mg bid‡	0.79 (0.66–0.95) p < 0.001 for non-inferiority, p = 0.01 for superiority	0.88 (0.66–1.17) p = 0.37	0.89 (0.80–0.99) p = 0.047

* Patients with creatinine clearance 30–49 ml/min received rivaroxaban 15 mg od.

† This hazard ratio is for the as-treated safety population. The hazard ratio for myocardial infarction for the intention-to-treat population was not presented in ROCKET AF. All p-values are for superiority unless stated otherwise. All three trials express the end-point event rates as % per year.

‡ Patients with serum creatinine levels of > 1.5 mg/dl received apixaban 2.5 mg bid. AF, atrial fibrillation; bid, twice daily; CI, confidence interval; od, once daily; TIA, transient ischaemic attack.

**Table 2:** Overview of safety data from phase III trials investigating rivaroxaban, dabigatran or apixaban compared with warfarin for the prevention of stroke and systemic embolism in patients with AF

			Hazard ratio (95% CI)			Side effects occurring significantly more in study drug vs. warfarin (%)
				
Study	Drug	Discontinuation: study drug vs. warfarin (%)	Major bleeding	Intracranial bleeding	Gastrointestinal bleeding	
ROCKET AF (18)	Rivaroxaban 20 mg od*	23.7 vs. 22.2	1.04 (0.90–1.20) p = 0.58	0.67 (0.47–0.93) p = 0.02	1.45†	Epistaxis 10.14 vs. 8.55, p < 0.05; haematuria 4.16 vs. 3.40, p < 0.05
RE-LY (17)	Dabigatran 150 mg bid	21.2 vs. 16.6	0.93 (0.81–1.07) p = 0.31	0.40 (0.27–0.60) p < 0.001	1.50 (1.19–1.89) p < 0.001	Dyspepsia 11.3 vs. 5.8, p < 0.001
	Dabigatran 110 mg bid	20.7 vs. 16.6	0.80 (0.69–0.93) p = 0.003	0.31 (0.20–0.47) p < 0.001	1.10 (0.86–1.41) p = 0.43	Dyspepsia 11.8 vs. 5.8, p < 0.001
ARISTOTLE (19)	Apixaban 5 mg bid‡	25.3 vs. 27.5	0.69 (0.60–0.80) p < 0.001	0.42 (0.30–0.58) p < 0.001	0.89 (0.70–1.15) p = 0.37	No breakdown of adverse events provided, but total adverse events occurred in almost equal proportions

* Patients with creatinine clearance 30–49 ml/min received rivaroxaban 15 mg od.

† Relative risk calculated from data in supplementary table; 224 bleeding events (3.2%) in rivaroxaban group compared with 154 events in the warfarin group (2.2%, p < 0.001).

‡ Patients with serum creatinine levels of > 1.5 mg/dl received apixaban 2.5 mg bid. AF, atrial fibrillation; bid, twice daily; CI, confidence interval; od, once daily. Definitions of bleeding: RE-LY: Major bleeding was defined as a reduction in the haemoglobin level of ≥ 2 g/dl, transfusion of ≥ 2 units of blood or symptomatic bleeding in a critical area or organ.

ROCKET AF: Major bleeding was defined as clinically overt bleeding associated with any of the following: fatal outcome, involvement of a critical anatomical site, fall in haemoglobin concentration > 2 g/dl, transfusion of > 2 units of whole blood or packed red blood cells or permanent disability. Non-major clinically relevant bleeding was defined as overt bleeding not meeting the criteria for major bleeding, but requiring medical intervention, unscheduled contact (visit or telephone) with a physician, temporary interruption of study drug (i.e. delayed dosing), or causing pain or impairment of daily activities.

ARISTOTLE: Major bleeding was defined as clinically overt bleeding associated with any of the following: fatal outcome, occurring at a critical site, decrease in the haemoglobin level of ≥ 2 g/dl or transfusion of ≥ 2 units of packed red cells. The secondary safety outcome was a composite of major bleeding and clinically relevant non-major bleeding, which was defined as clinically overt bleeding that did not satisfy the criteria for major bleeding and that led to hospital admission, physician-guided medical or surgical treatment, or a change in antithrombotic therapy.

When major bleeding occurs in patients taking warfarin, anticoagulation can be reversed by administering vitamin K and fresh frozen plasma. Specific antidotes for the new OACs do not exist and management is largely supportive, given the relatively short half-lives of these drugs (22). Nevertheless, this may be a source of concern that will influence clinicians' and patients' choice of anticoagulant.

Regular international normalised ratio (INR) monitoring is needed for optimal anticoagulation using warfarin, and this can be a source of concern and inconvenience for patients (14,23). The three new OACs are either once-daily (rivaroxaban) or twice-daily (dabigatran and apixaban) regimens that do not require routine anticoagulation monitoring.

The main side effects of the new OACs relate to minor bleeding events, as may be expected; these may not be dangerous, but could impact on patient quality of life. Rates of gastrointestinal bleeding were similar or higher for all three new OACs compared with warfarin. Rivaroxaban showed an increased incidence of haematuria and epistaxis in ROCKET AF (Rivaroxaban Once daily, oral, direct factor Xa inhibition Compared with vitamin K antagonism for prevention of stroke and Embolism Trial in Atrial Fibrillation) (18), and in RE−LY (Randomized Evaluation of Long-term anticoagulation therapY) dabigatran showed higher rates of dyspepsia (17) compared with warfarin. A breakdown of adverse events is not available for apixaban, but the ARISTOTLE (Apixaban for Reduction In STroke and Other ThromboemboLic Events in atrial fibrillation) trial investigators reported that the total was similar to that seen with warfarin (19). In the three trials, the discontinuation rates of the new OACs were similar to or higher than (in the case of dabigatran) the discontinuation rates observed in patients receiving warfarin; however, it is difficult to extrapolate from trial conditions what discontinuation rates would equate to in clinical practice. More patients taking dabigatran in the RE-LY trial had a myocardial infarction than those receiving warfarin (17), although a subsequent re-evaluation after detection of several additional primary efficacy and safety outcome events found that the difference was not statistically significant, with a revised relative risk of myocardial infarction in the dabigatran 150 mg twice-daily group of 1.27 (95% confidence interval 0.94–1.71; p = 0.12) compared with warfarin (24). Nevertheless, a recent meta-analysis involving trials of use of dabigatran for other indications suggested an increase in observed rates of myocardial infarction compared with the controls used (25). As noted above, comparisons between the different new OACs are not straightforward because the studies had different populations and designs, and there have been no head-to-head comparison trials. Therefore, in this article, we have emphasised differences between warfarin and the new OACs, rather than differences between these agents.

## Cost-effectiveness

We found five cost–benefit analyses for dabigatran, including a UK National Institute for Health and Care Excellence (NICE) technology appraisal, and for rivaroxaban, a NICE technology appraisal only (Table 3). Incremental cost-effectiveness ratio (ICER) estimates for dabigatran 150 mg compared with warfarin ranged from £5609 to £56,911 per quality-adjusted life-year (QALY) (26–29). The cost-effectiveness of dabigatran increased in groups more at risk of stroke, such as older patients: Freeman et al. (26) used the youngest patient group and estimated the second-highest ICER/QALY (for 150 mg twice-daily dose). Drug costs for dabigatran used in these analyses varied with a range of £1.99–£8.30 (based on 27 May 2012 exchange rates from http://markets.ft.com), with Shah et al. (28) and Freeman et al. (26) assuming the highest daily drug cost. The balance of cost-effectiveness is sensitive to the assumed cost of stroke treatment and follow up, which is much higher in the report of Sorensen et al. (29), which partly accounts for the much lower ICER estimate for dabigatran than in the other studies. The NICE technology appraisal for rivaroxaban found that it was likely to be cost-effective for adults with AF and one or more risk factors for stroke, with ICERs of less than £29,500/QALY (30).

**Table 3:** Cost-effectiveness analyses for dabigatran and rivaroxaban for the prevention of stroke and systemic embolism in patients with AF

			Incremental cost-effectiveness ratio (UK £/QALY)*			
			
Drug	Study	Population	Sequential regimen of 150 mg bid dabigatran before age 80 followed by 110 mg bid afterwards	Dabigatran 110 mg bid	Dabigatran 150 mg bid	Rivaroxaban 20 mg or 15 mg od
Dabigatran	Sorensen et al. (29)	Patients with AF and at ≥ 1 additional risk factor for stroke or impaired left ventricular ejection fraction. Mean CHADS_2_ score 2.1; mean age at starting 69 years		18,608	5609	
Dabigatran	Pink et al. (27)	Patients at moderate to high risk of stroke with AF and a baseline CHADS_2_ score of 2.1; mean age at starting 71 years		43,074	23,082	
Dabigatran	Freeman et al. (26)	Patients aged 65 years at starting with non-valvular AF and CHADS_2_ score of ≥ 1		32,710	28,970	
Dabigatran	Shah et al. (28)	Patients aged 70 at starting with AF at moderate risk of stroke (CHADS_2_ score of 1 or 2)		95,775	56,911	
Dabigatran	NICE technology appraisal guidance (31)	Population reflects that of RE-LY trial, i.e. adult patients with AF and ≥ 1 additional risk factor for stroke and eligible for anticoagulation	18,900			
Rivaroxaban	NICE technology appraisal guidance (30)	Population reflects that of ROCKET AF, i.e. adult patients with AF who were at moderate to high risk of stroke (CHADS_2_ score ≥ 2)				< 29,500

* Exchange rates based on 27 May 2012 rates from http://markets.ft.com

AF, atrial fibrillation; bid, twice daily; NICE, National Institute for Health and Care Excellence; od, once daily; QALY, quality-adjusted life-year.

The dabigatran technology appraisal by NICE considered the use of dabigatran in a two-tier regimen of 150 mg twice daily before the age of 80 followed by a switch to 110 mg twice daily after the age of 80 for stroke prevention in patients with AF and at least one additional risk factor for stroke. This strategy was found to be cost-effective for the UK National Health Service (NHS), with ICERs of less than £18,900/QALY in patients starting treatment younger than age 80, assuming a monitoring cost of £241.54 per annum for warfarin (31). The original manufacturer's submission to NICE estimated an INR cost of £414.90: this was based on the assumption that dabigatran would completely replace warfarin, so includes the fixed overheads of running anticoagulation clinics. The monitoring cost per patient used in the NICE final appraisal determination was estimated using the NICE AF costing report from 2006 (32) and the NHS reference costs for 2008/09 (33), both inflated to 2009/10 prices, but because dabigatran would be unlikely to completely replace warfarin, this figure was reduced to account for the fixed costs of running anticoagulation clinics (34).

As we performed our search, three further cost-effectiveness analyses have been published comparing warfarin with dabigatran in the general AF population, set in Denmark (35), Sweden (36) and the UK (37). All concluded that dabigatran was cost-effective in comparison with warfarin, with an ICER of €7000 per QALY in the Danish study (35), €7700 in the Swedish study (36) and approximately £4800–£7000 per QALY in the UK study (37). There has also been a US cost-effectiveness study that compared rivaroxaban with warfarin, and showed rivaroxaban to be cost-effective with an ICER of $27,500 per QALY (38).

Warfarin has previously been found to be cost-effective for stroke prevention in patients with AF (39,40). This was confirmed in an older age group by a recent economic evaluation conducted alongside the BAFTA trial (41). Cost-effectiveness for warfarin is dependent on the level of INR control achieved. Good INR control can be achieved in routine care with values within the therapeutic range around 68% of the time (42,43). The NICE technology appraisal for dabigatran estimated an ICER cost of £47,000/QALY for the subset of patients with the best INR control, although this figure was substantially reduced if the higher INR monitoring costs of £414.90 were assumed (31). There is a substantial group of patients for whom anticoagulation is indicated, but who prefer not to take warfarin. The ACTIVE A (Atrial fibrillation Clopidogrel Trial with Irbesartan for prevention of Vascular Events) trial used a population of patients with AF who were deemed unsuitable for vitamin K antagonist therapy; however, one of the main reasons for this was patient preference not to take warfarin, which was the case in approximately one in four patients enrolled (44). In these patients, new OACs are likely to be highly cost-effective compared with using an antiplatelet agent or no therapy.

## Implications for primary care

The new OACs offer genuine alternatives to warfarin and exhibit similar or better efficacy, better safety in some parameters (e.g. intracranial haemorrhage) and are easier to use. The safety data from the trials show a profile of non-inferiority to warfarin, but drugs that have appeared to have a good safety profile in clinical trials in the past have later been withdrawn (45–47), whereas warfarin is a well-established drug that can be difficult to use. Key determinants of cost-effectiveness are quality of INR control and cost of INR monitoring on warfarin (14,29). Different models of INR monitoring have different costs, for example warfarin dosing in primary care costs less than in secondary care (32).

**Figure 1:**
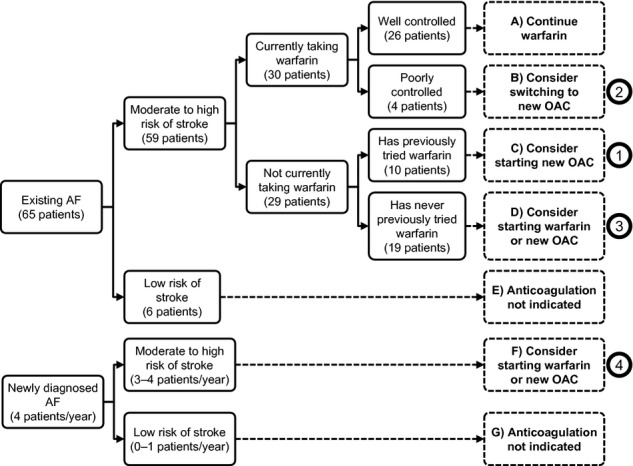
Quantifying workload in general practice for introducing new anticoagulants by different categories of AF patient. Patients were categorised into seven groups (A–G), and assigned an order of priority (1–4) for receiving a new OAC. The following assumptions were made: the average General Practice population size in England is 6600 (54), AF has a population prevalence of approximately 1% (3), the incidence of newly diagnosed cases of AF is 0.6 per 1000 (55), approximately 90% of patients with AF are at high or moderate risk of a stroke/transient ischaemic attack using the CHADS_2_ score (50); approximately 47% of patients who should be receiving warfarin are not (58); patients with CHADS_2_ scores ≥ 2 are not receiving warfarin (59,60), 88% of UK participants have a mean time in therapeutic range of > 65% (49); discontinuation rates are > 25% in the first year for patients with AF started on warfarin (62). AF, atrial fibrillation; OAC, oral anticoagulant.

The NICE technology appraisals have major potential implications for primary care because they support the use of dabigatran and rivaroxaban in most circumstances. However, even if new OACs are cost-effective, this does not mean that they are affordable and total cost may be more important to local commissioning groups than cost-effectiveness, making the introduction of a new drug problematic in the current economic environment. For example, one costing study suggested that the annual cost of anticoagulation is about seven times higher with dabigatran than it is with warfarin (48). With this in mind, we have divided the general practice AF population into seven groups according to risk of stroke and previous experience of warfarin, and prioritised these groups in order of cost-effectiveness of prescribing a new OAC. We have quantified the number of people who would be in each group for an average-sized practice (Figure 1). This would suggest that for a typical practice of 6600 people, starting a new OAC should be considered in 14/65 (22%) of patients on the AF register – i.e. in those people who have previously tried warfarin, or in those for whom the INR control is poor. It is worth noting that we have assumed that 88% of patients have good INR control, which is based on information from the ACTIVE-W trial (49). It is likely that this figure would be lower in the general population (49). A further 19/65 (29%) people not receiving warfarin should be considered for either a new OAC or warfarin (Figure 1).

A patient's risk of stroke can be quantified using CHADS_2_ or CHA_2_DS_2_-VASc scores (Table 4) (50,51). Previously, patients were stratified into high-, medium- or low-risk groups, with only patients in the high-risk group (CHADS_2_ score ≥ 2) recommended to receive anticoagulation (1). However, there is a reduction in stroke risk from anticoagulation even for patients with only one risk factor for stroke (52). We are now moving towards a binary system involving consideration of anticoagulation in patients with a CHADS_2_ score of ≥ 1 if the CHA_2_DS_2_-VASc score is ≥ 1. This system results in better identification of a low-risk group for whom the risk of stroke is small (Table 5). We note, however, that while the NICE technology appraisal recommends dabigatran for patients with AF and one or more additional risk factors for stroke, neither female sex nor peripheral vascular disease constituted one of the risk factors (31) (both are included as additional risk factors in the CHA_2_DS_2_-VASc score, Table 4). For the purposes of this review, we have defined moderate to high risk as the presence of one additional risk factor for stroke and assumed consideration of anticoagulation in these individuals, with the benefits of anticoagulation increasing as risk of stroke increases.

**Table 4:** Comparison of CHADS_2_ and CHA_2_DS_2_-VASc stroke risk scoring systems

Risk factor	CHADS_2_ score (50)	CHA_2_DS_2_-VASc score (51)
Congestive heart failure	1	1
Hypertension	1	1
Age ≥ 75 years	1	2
Diabetes mellitus	1	1
Previous stroke or TIA	2	2
Vascular disease	–	1
Age 65–74	–	1
Female sex category	–	1

TIA, transient ischaemic attack.

**Table 5:** Rates of thromboembolism that resulted in hospital admission and death at 1-year follow up, from a Danish cohort study of 73,538 patients with atrial fibrillation not receiving treatment with a vitamin K antagonist (64)

CHADS_2_ score	Rate of significant thromboembolism per 100 person-years (95% CI)	CHA_2_DS_2_-VASc score	Rate of significant thromboembolism per 100 person-years (95% CI)
0	1.67 (1.47–1.89)	0	0.78 (0.58–1.04)
1	4.75 (4.45–5.07)	1	2.01 (1.70–2.36)
2–6	12.27 (11.84–12.71)	2–9	8.82 (8.55–9.09)

CI, confidence interval.

The 2011 consensus statement on dabigatran from Health Improvement Scotland advised continued first-choice use of warfarin in patients with AF who are at moderate or high risk of stroke and have good INR control, and for dabigatran or rivaroxaban to be used in those with poor INR control despite good adherence or an allergy to warfarin (53). Making a recommendation for newly diagnosed patients with AF who have an indication for an OAC agent is less clear cut; Health Improvement Scotland recommended that all patients should try warfarin first line, but NICE advises that dabigatran or rivaroxaban is likely to be cost-effective and that either of these two new OACs or warfarin can be prescribed after a discussion of risks and benefits. Individual factors such as preference for ease of coagulation monitoring, renal function, a risk of falls or history of dyspepsia will influence the decision, which should be an informed choice made jointly by the patient and general practitioner (GP). We have recommended considering a new OAC in groups C, B, D and F in Figure 1, in the order indicated.

**Assumptions made to arrive at the numbers for the different population groups in Figure 1**

Average GP population size is 6600 in England (54)

AF has a population prevalence of approximately 1% (3,4)

The incidence of a new case of AF is 0.6 per 1000 (55); this has been estimated at 1.7 per 1000 for chronic AF in general practice (56) and 2.9 per 1000 for all cases of AF (57)

Approximately, 90% of patients with AF are at high or moderate risk of a stroke/transient ischaemic attack using the CHADS_2_ score (50). This number may be slightly less if only patients with a CHADS_2_ score of ≥ 2 were included

A 2006 costing report by NICE estimated that 47% of patients who should have been receiving warfarin were not, based on an assumption that all high-risk and half of moderate-risk patients with AF should be on anticoagulation (58). More recent NHS improvement audits and a study of the QResearch database have found that similar percentages of patients with CHADS_2_ scores of ≥ 2 are not receiving warfarin (59–61). If we include all patients with ≥ 1 additional risk factor for stroke, it is likely that no more than 50% will be receiving warfarin

In a *post hoc* analysis of patients on warfarin in the ACTIVE-W trial, 88% of UK participants had a mean time in therapeutic range of > 65%; this figure may be lower in the general population (49)

Fang et al. (62) found discontinuation rates of more than 25% in the first year for patients started on warfarin for AF in the ATRIA (AnTicoagulation and Risk factors In Atrial fibrillation) cross-sectional study. Assuming this rate of discontinuation and that 30 patients per GP are currently taking warfarin, 10 of the patients currently not taking warfarin at a GP surgery may have previously tried it. In the AVERROES (Apixaban VERsus acetylsalicylic acid to prevent stROkES) trial, which compared apixaban with acetylsalicylic acid in patients with AF with an additional risk factor for stroke but deemed unsuitable for warfarin, 40% of patients had previously tried a vitamin K antagonist, which helps corroborate our estimate (63)

## Conclusions

Anticoagulant prophylaxis with warfarin is highly effective in reducing the risk of thromboembolic stroke risk in patients with AF; however, it continues to be underused for a variety of reasons, including patient and physician reluctance to initiate treatment, and problems incurred while on treatment. The availability of new OACs as cost-effective alternatives to warfarin will mean that in the future a higher proportion of people with AF and at high risk of stroke can receive effective stroke prevention medication.
